# Establishing a New Patient-Specific Implantation Technique for Total Ankle Replacement: An In Vitro Study

**DOI:** 10.1177/19386400211029741

**Published:** 2021-07-12

**Authors:** Leif Claassen, Philipp Luedtke, Dennis Nebel, Daiwei Yao, Sarah Ettinger, Kiriakos Daniilidis, Christina Stukenborg-Colsman, Christian Plaass

**Affiliations:** DIAKOVERE Annastift, Orthopädische Klinik der Medizinischen Hochschule Hannover (MHH)—Hannover Medical School; DIAKOVERE Annastift, Orthopädische Klinik der Medizinischen Hochschule Hannover (MHH)—Hannover Medical School; Laboratory for Biomechanics and Biomaterials of the Hannover Medical School; DIAKOVERE Annastift, Orthopädische Klinik der Medizinischen Hochschule Hannover (MHH)—Hannover Medical School; DIAKOVERE Annastift, Orthopädische Klinik der Medizinischen Hochschule Hannover (MHH)—Hannover Medical School; Hannover, Germany; and OTC Regensburg, Regensburg, Germany; DIAKOVERE Annastift, Orthopädische Klinik der Medizinischen Hochschule Hannover (MHH)—Hannover Medical School; DIAKOVERE Annastift, Orthopädische Klinik der Medizinischen Hochschule Hannover (MHH)—Hannover Medical School

**Keywords:** ankle joint, talocrural joint, joint axis, total ankle replacement, CT scan, patient-specific implantation

## Abstract

**Background:**

Revision rates after total ankle replacements (TARs) are higher compared with other total joint replacements. The present study aimed to establish a new patient-specific implantation (PSI) technique for TAR.

**Material and methods:**

A total of 10 complete Caucasian cadaver legs had whole leg computed tomography scans. The individual geometrical ankle joint axis was determined, and based on this axis, the position of the prosthesis was planned. We assessed prosthesis placement, guiding block position, and preoperative and postoperative ankle rotational axes.

**Results:**

The guiding block position interobserver reliability was 0.37 mm 0.45 (mean ± SD) for the tibial guiding block. The value for the first talar guiding block was 1.72 ± 1.3 mm and for the second talar guiding block, 0.61 ± 0.39 mm. The tibial slope as well as the frontal angles of the anatomical tibial axis compared to the tibial and talar articular surfaces showed no statistically relevant differences with numbers available. The deviation of the assessed preoperative joint axis to the postoperative joint axis was 14.6° ± 7.8.

**Conclusion:**

The present study describes the results of an establishing process of a new PSI technique for TAR. The reliability of guiding block positioning and, thereby, prosthesis placement is sufficient.

**Level of Evidence:**

*Biomechanical study*

“A detailed knowledge of ankle joint anatomy and biomechanics is critical to optimize treatment.”

## Introduction

Ankle joint osteoarthritis is a limiting disease that affects about 1% of the population.^1^ In severe cases and after failed conservative treatment, the primary treatment alternatives are ankle joint arthrodesis and total ankle replacement (TAR).^2^ The number of performed TARs is still rising.^3,4^ Despite improvements, the revision rates are relatively high after TAR, ranging from 6.7% to 27.3%,^5-14^ whereas in recent studies, survival rates after transfibular TAR of 100% after 2 years in 159 cases and 98.9% in a prospective study including 89 cases could be reached.^15,16^ Revision rates after TAR are tendentiously higher compared to ankle fusion and also compared to total hip or knee joint replacement.^17-19^ One widely discussed reason is a nonanatomical prosthesis placement. Still, there is no consensus about the ankle joint axis.^20-32^ A detailed knowledge of ankle joint anatomy and biomechanics is critical to optimize treatment.^33-35^ The relevance of ankle joint anatomy and biomechanics for prosthesis placement is evident.^36-39^

In total knee replacement, the development of patient-specific implantation (PSI) techniques evolved in recent years. Although previous studies could not prove a general superiority of PSI techniques compared to conventional implantation techniques, the development process of PSI techniques amplified the discussion about prostheses placement and orientation.^40-45^ A PSI technique is already established for TAR with promising results regarding accuracy of implant placement.^36,46^ However, there is no information and discussion of the prosthesis placement, especially regarding the joint axis.^36,46,47^ Recently, Claassen et al^48,49^ described basic anatomical parameters of the ankle joint and a suggestion for the determination of the ankle joint axis.

The aim of the present study was the establishment and evaluation of a new system for patient-specific TAR implantation.^49^

## Material and Methods

### Cadaver

The present study was based on 10 full fresh frozen Caucasian cadaver lower extremities, including the femoral head. We excluded specimens with severe deformity, severe degenerative changes in any joint of the leg, or obvious previous trauma. Severe degenerative changes were defined as a Kellgren and Lawrence Score >3 based on the Kellgren and Lawrence criteria, including osteophytes, subchondral sclerosis, and cysts.^50^ Signs of previous trauma would have been fracture lines, callus formation, deformities, or cortical inhomogeneities.

### Imaging and Data Processing

Each cadaver lower extremity received the following prior to and after prosthesis implantation: a computed tomography (CT) scan, X-ray of the ankle in 2 planes, and a conventional X-ray of the whole leg. CT scans were performed with a slice thickness of 0.63 mm, including 10 cm around the femoral head, 10 cm proximal and 10 cm distal of the knee joint center, and 10 cm proximal and 10 cm distal of the ankle joint center, including the whole foot. CT scans were integrated into Mimics (Materialise, Leuven, Belgium) and resliced applying the coordinate system recommended by the International Society of Biomechanics (ISB).^51^

**Figure 1.**
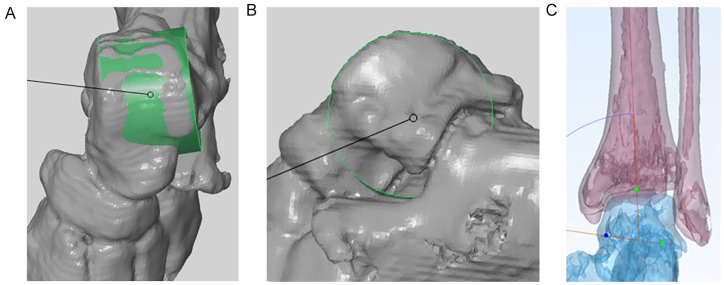
Geometrical ankle joint axis. (A) and (B), dorsoplantar and lateral views of the CT-based generated 3D model of the talus with the foot. The talar articular ankle joint surface was marked, and a best-fitting cone (green) was generated with GOM Inspect. The axis of the cone was defined to be the patient’s individual geometrical ankle joint axis. (C) The cone axis/geometrical ankle joint axis (talar red line) is illustrated compared to the tibial mechanical axis (tibial red line). The geometrical ankle joint axis was the base for planning of prosthesis placement.

After segmentation also with Mimics we generated 3-dimensional (3D) models of the talus with foot as well as of the tibia with fibula. The 3D models of the talus and foot were transferred to GOM Inspect (Version 8.0, Gesellschaft für optische Messtechnik, Braunschweig, Germany). This software enables the generation of a best-fitting cone to the talar articular surface of the ankle joint.^49^ We defined the talar articular surface as the area between the transition from concavity and convexity from the talus dome to processus posterior tali posteriorly and talus neck anteriorly. The shoulders of the talus dome defined the medial and lateral borders of the talar articular surface. The axis of the cone was defined to be the geometrical ankle joint axis (Figure 1).

### Planning of Prosthesis Implantation

**Figure 2.**
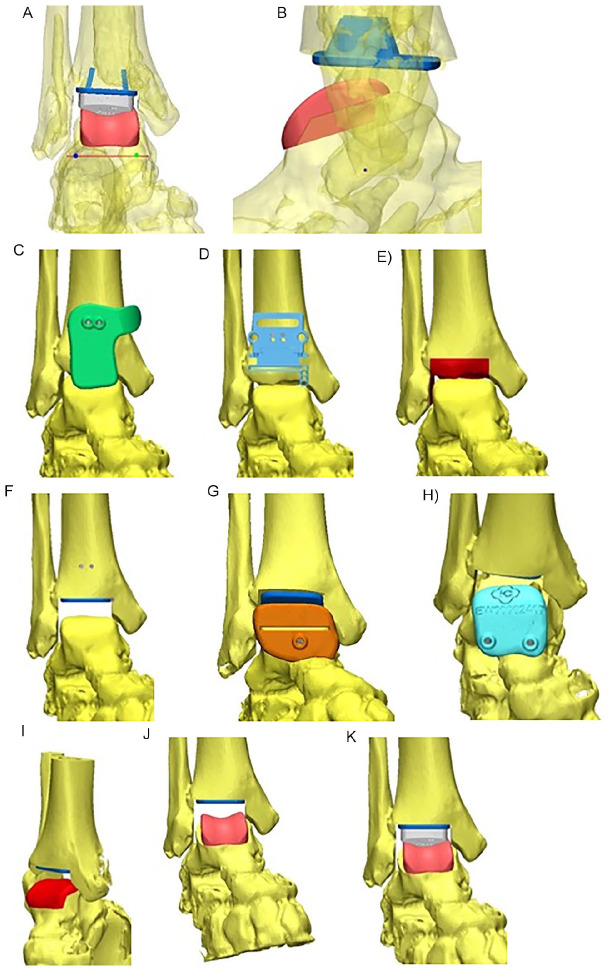
Planning of prosthesis placement: A. The individual geometrical ankle joint axis is illustrated in a frontal view in red. It is the base for the aimed prosthesis position. The final prosthesis placement is also illustrated with tibial and talar components and the polyethylene. The talar prosthesis component was placed in each plane, including rotation in the axial plane oriented to the geometrical ankle joint axis. The tibial prosthesis component was placed in orientation to the anatomical tibial axis. B. The aimed prosthesis position is illustrated in a sagittal view. Also, in this plane, the geometrical ankle joint axis (visible as a dot) was the base for prosthesis placement. C. The tibial guiding block was generated aiming to provide a distinct fit to the tibia. It enabled insertion of 2 pins. D. After removing of the guiding block, the conventional cutting block of the prosthesis was applied in a determined direction to perform the tibial saw cuts. The slope and the distal/proximal translation were defined preoperatively. E. The planned amount and orientation of tibial resection is visualized. F. The aimed placement of the tibial prosthesis component is visualized. G. The first talar guiding block is illustrated. It is an actual cutting block to provide the first talar saw cut. H. The second talar guiding block enables insertion of 2 pins that served as the guidance for the application of the conventional cutting blocks in the determined position. I. The amount and orientation of talar resection is visualized. (J) and (K) The aimed prosthesis position, including the polyethylene is visible.

The prosthesis placement was planned via computer based on the determined geometrical ankle joint axis. The talar prosthesis component was oriented regarding rotation in the axial plane and varus/valgus angle to the geometrical ankle joint axis. The tibial component placement was oriented to the anatomical tibial axis. The PSI guiding blocks were made computer based depending on the planned prosthesis position (Figure 2). The implant size was determined by the tibial width. For the specific implant used in this study, different lengths of the tibial implant were not available, and an imperfect coverage of the bone surface especially in anterior-posterior direction had to be accepted.

### Prosthesis Implantation With Assessment of Block Placement and Biomechanical Ankle Joint Axis

The leg was fixed to the bench, enabling free mobilization of the ankle joint and foot. For the assessment of block placement and biomechanical ankle joint axis, we used an infrared-marker tracking system (Polaris P4, Northern Digital Inc, Waterloo, ON, Canada) and a custom-made software written in Microsoft Visual Studio (Microsoft, Redmond, WA). To adjust the leg to the CT scan, the proximal and distal tibial articular surfaces as well as the talar articular surface were tracked. Additionally, we implanted 1 marker in the distal tibia and 1 marker in the talar head after ensuring that marker placement did not affect bony preparation and prosthesis implantation. Prior to skin incision, the biomechanical ankle joint axis was assessed independently by 2 investigators 3 times each by manually moving the ankle joint repeatedly from 10° dorsiflexion to 30° plantarflexion. Doing this via Polaris, the relative movements of the talar and tibial markers were recorded, indicating the joint axis. This procedure was repeated after prosthesis implantation. This provided the individual biomechanical preoperative and postoperative ankle joint axes and served as comparison to the individual geometrical ankle joint axis described above.

**Figure 3.**
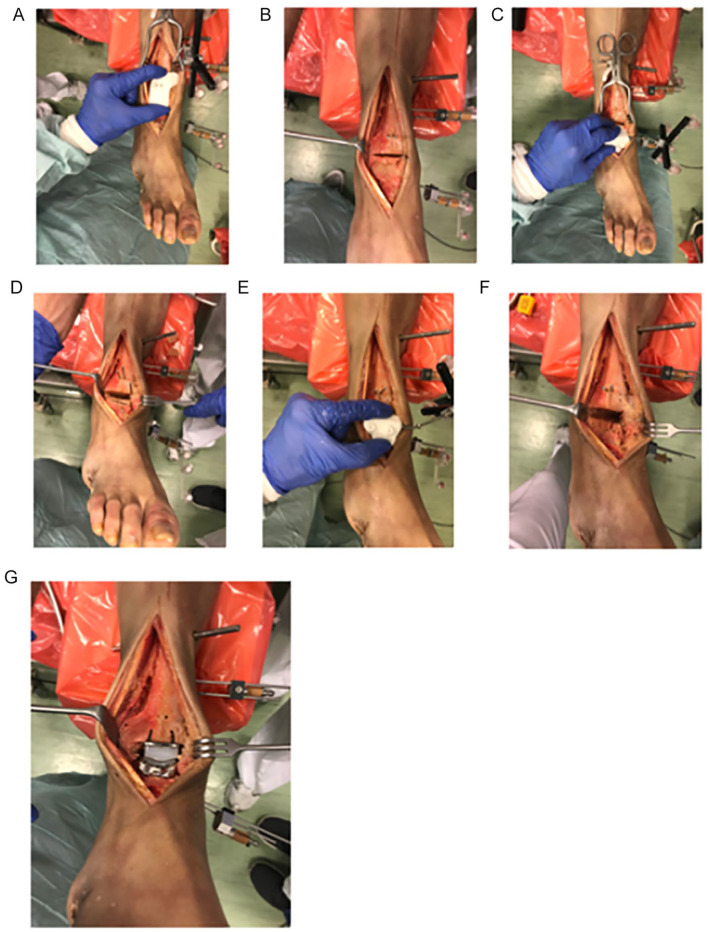
Prosthesis implantation: A. Application of the tibial guiding block. B. The tibia saw cut is done. C. Application of the first talar guiding block. D. The first talar saw cut is done. E. Application of the second talar guiding block. F. The talar preparation is finished. G. The prosthesis implantation is finished.

The used prosthesis was TARIC (Implantcast GmbH, Buxtehude, Germany) and the implantation basically followed the implantation guide. During TAR implantation, we used the individually planned and individually generated guiding blocks that were illustrated in Figures 2C, 2G, and 2H. Whereas the first talar saw block enables a sawcut and thereby is a real saw block, the tibial and second talar blocks guided pins. With these pins, conventional instruments could be applied and saw cuts were performed in the planned position with conventional instruments. That is why we rather use the term guiding block than saw block. During preparation, each guiding block was applied independently by 2 investigators 3 times each, with determination of the respective placement. After applying the tibial guiding block, the 2 guiding pins were inserted. The placement of the pins was controlled with the conventional instruments. The resection could be correlated to the planned resection. The orientation of the talar prosthesis placement was determined via the first and second talar guiding blocks. The consecutive preparation steps followed the implantation guide. Again, the resection could be correlated to the planned resection (Figure 3).

### CT Scan and X-ray Evaluation

**Figure 4.**
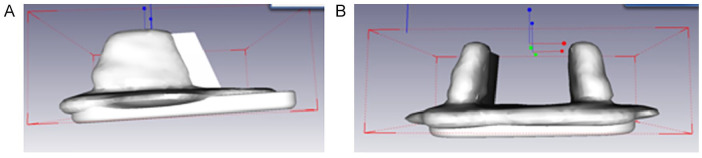
Assessment of prosthesis placement based on computed tomography scans: A. Sagittal plane. B. Frontal plane. A coordinate was applied to the planned prosthesis component and to the implanted prosthesis component. Thereby, the translational and angular deviations are evaluated.

The preoperative CT scan was used for planning of prosthesis placement. Using the postoperative CT scan a comparison of the actual tibial and talar prosthesis position to the planned position was done. We analyzed the proximal/distal, medial/lateral, and anterior/posterior translations and the varus and valgus tilt as well as the tilt in the sagittal plane. Additionally, the rotation of each prosthesis component in the axial position was assessed (Figure 4). For this evaluation, the CT scans were segmented with Amira (ThermoFisher Scientific, Waltham, MA), and the coordinate systems were generated with GOM Inspect for matching the final surface (Gesellschaft zur Förderung angewandter Informatik, Berlin, Germany).

**Figure 5.**
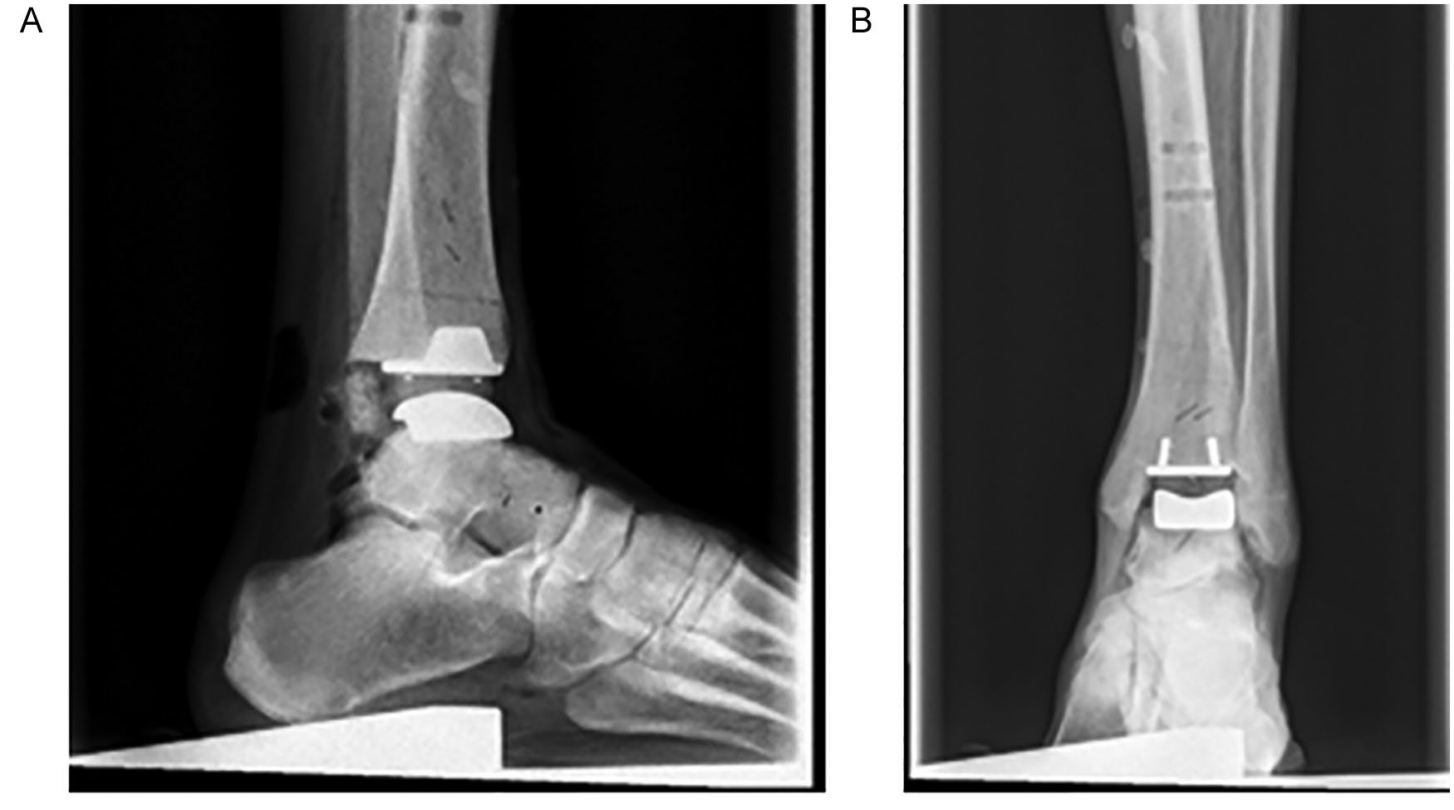
Assessment of prosthesis placement based on X-ray: A. Sagittal plane. B. Frontal plane. The prosthesis placement was assessed compared to the anatomical tibial axis.

In conventional X-ray, we analyzed the tibial and talar articular angles as well as the tibial slope in comparison to the anatomical tibial axis as published before.^52,53^ We determined the distal medial tibial articular angle as the angle of the anatomical tibial axis to the distal tibial articular surface and the angle of the anatomical tibial axis to the talar articular surface, both based on anterior-posterior radiographs. Additionally, based on lateral radiographs, we determined the tibial slope as the angle of the anatomical tibial axis and the line connecting the anterior and posterior edges of the distal tibial articular surface (Figure 5).

### Statistical Analysis

Statistical analysis was performed with IBM SPSS Statistics (Version 24.0, IBM, Armonk, New York, NY). A Wilcoxon matched-pair test was used for data on the interval scale because we had nonparametric related samples. The values are expressed as means with SDs, with *P* <.05 being taken to be significant. For intrarater deviation, we assessed the Pearson correlation coefficient for parametric data and the Spearman correlation coefficient for nonparametric data.

## Results

During implantation, we saw the following adverse events. In 3 specimens, not enough bone was resected; tibial and tibial aftercuts had to be done in 2 and deltoid release in 2 instance. Because of the instability, we chose a 3 mm higher polyethylene in 1 case. In 4 cases, the medial malleolus was fractured with necessary refixation via screws, which was anatomically possible in all cases.

In 2 tibial guiding blocks and in 1 talar block, the fit to the bone was judged unstable. Still, a reliable position could be found. The bone cuts were performed with the cutting blocks placed in the best-fitting position. For each cutting block, we found a high interobserver reliability. Except for the first talar cutting block, the interobserver deviation was lower than 1 mm (Table 1). X-ray evaluation showed no statistically significant differences regarding the distal tibial medial angle, tibial slope, and medial angle of the talar articular surface compared to the tibial anatomical axis, regarding the preimplantation measurements and the later tibial component position with numbers available (Table 2). The results of geometrical and biomechanical preoperative and postoperative ankle joint axes are shown in Table 3. The CT-based assessment of prosthesis placement was possible only in 5 of 10 cases with sufficient accuracy because of metallic artifacts, despite the fact that CT scans were performed with artifact suppression. Table 4 shows the deviation of implanted prosthesis components in comparison to the planned position.

**Table 1.** Cutting Block Reliability.^a^

Interobserver reliability	Mean	SD	Correlation coefficient	*P* value
Tibial cutting block (mm)	0.37	0.45	1.0	<.001
First talar cutting block (mm)	1.72	1.3	1.0	<.001
Second talar cutting block (mm)	0.61	0.39	1.0	<.001

a The interrater difference of the tibial cutting block and the second talar cutting block was <1 mm. The mean deviation of the first talar cutting block was slightly higher but still <2 mm.

**Table 2.** X-ray Evaluation.^a^

	Preoperative		Postoperative		
	Mean	SD	Mean	SD	*P* value
Distal tibia medial angle (degrees)	87.9	2.5	85.4	3.1	NS
Medial angle of talar articular surface vs tibial anatomical axis (degrees)	88.1	3.1	87.8	5.2	NS
Tibial slope (degrees)	6.5	3.6	5.7	4.1	NS

a The evaluation of the prosthesis positioning via X-ray included the tibial slope, and the angles of the tibial anatomical axis compared the tibia or talar articular surfaces. There were no statistically relevant differences between the preoperative and postoperative positions, with numbers available.

**Table 3.** Joint Axis Assessment.^a^

Joint axis assessment	Mean	SD	95% CI
Planned axis vs preoperative axis (degrees)	18.8	9.1	14.5-23.1
Planned axis vs postoperative axis (degrees)	17.1	10.1	12.4-21.9
Preoperative axis vs postoperative axis (degrees)	14.6	7.8	10.9-18.2

a The deviations of the planned to the preoperative and postoperative axes as well as the deviation of the preoperative to the postoperative axis was >10°. Also, because the lower border of the 95% 95% CIs are higher than zero, this indicates a significant difference of each compared axis.

**Table 4.** Deviation of Implanted Tibial and Talar Prosthesis Components to Preoperative Planning.^a^

		Translation			Rotation		
		Anterior/Posterior (mm)	Medial/Lateral (mm)	Proximal/Distal (mm)	Valgus/Varus (degrees)	Extension/Flexion (degrees)	External/Internal rotation (degrees)
Tibial prosthesis component	Mean ± SD	−1.7 ± 3.0	0.6 ± 1.2	1.7 ± 4.5	−0.5 ± 8.5	−0.3 ± 6.8	2.4 ± 5.2
	95% CI	−3.8 to 0.4	−0.7 to 1.9	−3.0 to 6.5	−9.4 to 8.3	−7.4 to 6.9	−3.1 to 7.9
	*P* value	<.05	NS	<.05	<.05	<.05	<.05
Talar prosthesis component	Mean ± SD	−2.0 ± 2.8	0.6 ± 3.4	1.6 ± 1.7	6.5 ± 12.7	−9.4 ± 3.1	7.0 ± 10.5
	95% CI	−5.5 to 1.4	−3.6 to 4.7	−0.5 to 3.7	−9.3 to 22.3	−13.3 to −5.5	−6.0 to 20.0
	*P* value	<.05	<.05	<.05	<.05	<.05	<.05

a This evaluation was possible in 5 of 10 cases. This analysis is a comparison of the planned prosthesis position to the position in postoperative computed tomography scans. Referred to the acceptable borders of 2 mm translation and 3° rotation from Berlet et al,^36^ only the mediolateral translation of the tibial component was within these borders. Positive mean values indicate a deviation in the first mentioned direction (eg, anterior), and negative mean values indicate a deviation in the second mentioned direction (eg, posterior).

## Discussion

This study evaluates a new system for patient-specific TAR implantation. Its aim was to place the prosthesis components in a physiological position by preoperative evaluation of the joint geometry. To achieve a high accuracy of component positioning, patient-specific cutting guides were evaluated.

The planning process was based on CT scans because the ankle joint cartilage layer thickness is low, with 1.1 mm for the talar articular surface and 1.16 mm for the tibial articular surface.^54^ Additionally, CT scans have the advantages of illustrating bony configuration in detail, wide availability, and lower susceptibility to movement artifacts compared with MRI.^54^

Established products of Materialise were used for segmentation and analyses, similar to previous studies.^22,24,36,43,44,55-57^ Therefore, reslicing of CT scans with respect to the coordinate system and generation of 3D bone models were done.

The present study showed a high reliability for guiding block positioning, with a total deviation <2 mm for all guiding blocks and <1 mm for 2 of 3 cutting blocks. The only comparable data for the ankle joint were published by Berlet et al.^36^ They reached a guiding block deviation <1 mm.^36^ For the knee joint too, to our best knowledge, further comparable data regarding accuracy of guiding block positioning are lacking. However, published data regarding final prosthesis placement after PSI of total knee arthroplasty is at least ambiguous. On the one hand, some authors found a high accuracy of postoperative alignment and prosthesis placement.^43,58^ On the other hand, the guiding blocks needed to be reapplied intraoperatively because of insufficient fit in up to 26.5%, and a meta-analysis revealed that in up to 36.4%, the PSI was stopped intraoperatively because of a poor match from planned prosthesis placement to intraoperative observations.^41,42^ Hence, the accuracy of guiding block positioning found seems to be acceptable. The implantation planning in this study was done on physiological ankles without deformities. In arthritic ankles with possible posttraumatic deformities, planning and achieving an anatomical position can be more challenging. Nevertheless, we think that with PSI techniques, a precise positioning of the implant is possible. With increased understanding of implant positioning, a more physiological position of the TAR should become possible.

Our results describe the reconstruction of preoperative articular surfaces because the tibial slope as well as the frontal angles between the anatomical tibial axis and the respective tibial and talar articular surfaces showed no relevant differences, preoperative to postoperative. In the used specimens with physiological joint geometry, all differences were <3°. This compares with Berlet et al^36^ showing component deviations <3°. Hsu et al^46^ published consecutive clinical data analyzing preoperative and postoperative radiographs of TAR implanted with patient-specific guiding blocks. They described deviation of the preoperative to postoperative alignment <3° in the coronal and sagittal planes. The distinct postoperative coronal alignment was 0.1° ± 0.9° varus and 0.0° ± 0.4° plantarflexion. In another consecutive prospective clinical study, Daigre et al^47^ analyzed the tibial component alignment. The coronal alignment was 1.8° ± 1.5° and the sagittal alignment, 2.4° ± 1.7°. The aimed rotational deviation of <3° was reached in 79.5% of all cases.

No previous study evaluated whether an anatomical joint axis can be restored with the implantation of a TAR. Moreover, there is no information as to whether the anatomical joint axis should be restored at all. There is no definition of the optimal axis for TAR implantation. Previous studies showed a wide variety of the, in this study used, geometrical axis of the ankle.^49^ Already, the preoperatively measured biomechanical axis differed from the geometrical axis, as did the postoperative axis despite precise placement of the cutting guides. Most actual TAR designs use a nonanatomical cylindrical shape of the talar component. This might be one reason why we have not been able to reconstruct the anatomical axis. On the other hand, we found a relevant difference between the geometrical and biomechanical axes. Because the ankle joint is generally thought to be a high congruent joint and because the joint axis is driven by the bony structures, a high correlation was expected prior to the study. Nevertheless, the incongruency of previous studies regarding analysis of the joint axis and the finding of this study suggest that the ankle joint axis is more complex than expected.^20,22,23,25-32,48,49,59-61^

With the present study, we suggest a new system for PSI of TAR. This process included a learning curve especially regarding planning and construction of guiding block configuration. In this specific implant, during planning, care must be taken that a lesion of the medial malleolus is avoided. The resection height of the tibia must be considered also in regard to the PE height. Regarding the guiding blocks, 2 aspects must be simultaneously respected: their design must allow a normal surgical approach, and their bony contact area must ensure a stable and distinct block placement.

The question of whether PSI techniques lead to enhanced results cannot be answered yet. Several studies regarding PSI of total knee arthroplasty could not prove a benefit for the patient in general.^40-45^ For TAR, there is only 1 system established for clinical use currently. Although the system allows a high precision of TAR placement, there are no data as to whether the patients have any profit of this technique. The main difference between the established product and that in the present study was that in the present study, the prosthesis placement was based on a supposed individual ankle joint axis. The established system primarily plans the prosthesis placement based on the mechanical tibial axis.^36^ The advantage of the present study is that with the detailed description of the methods and the development process, our practice can be translated to other prostheses designs.

The discussion about this topic highlighted the need of further evaluation of basic anatomical and biomechanical principles of the ankle joint. We need to define the healthy ankle joint axis and develop a sufficient and clinically available tool to reconstruct it. Evaluations are pending regarding which structure determines the ankle joint axis and whether there are bony structures as supposed or with relevant part ligaments. Mechanical versus kinematic alignment is not yet discussed for TAR. Additionally, information must be collected regarding the effect of changing the alignment via the operation: for example, should a valgus hindfoot axis be left in valgus or should a valgus ankle joint line be left in valgus.

Our study has some limitations. There is a low number of cases. Thus, a learning curve at the beginning of this study might have affected the results and resulted in adverse events such as fracture of the medial malleolus. With more cases and more experience, especially for the planning process, these adverse events can be avoided. The CT scans and conventional X-rays were taken non–weight-bearing. The evaluation of the ankle joint axis was performed manually. Maybe the use of a robot would have led to a more standardized movement and, thereby, a higher accuracy of testing results.^62^ Additionally, the movement axis was not itemized to PE movement to the tibial and talar components. Hence, the PE translation could have affected the joint axis. In clinical application, because the actual planning process itself is based on non–weight-bearing CT scans, weight-bearing radiographs should be performed prior to surgery to consider and analyze any foot deformities. In future, with broader availability of weight-bearing full-leg-length CTs, these probably will be used for the planning of implant position.

The strengths of the present study are the evaluation and presentation of a new system for patient-specific TAR implantation, including the joint axis. Previous studies regarding this topic focused on accuracy of guiding block and implant placement. The possibility of both high accuracy of guiding block and implant positioning is supported by this study.

## Conclusion

This study describes the establishing of a new system for patient-specific TAR implantation. Patient-specific cutting guides can be reliably placed where they were designed to go. This study supports the discussion about where and how to position TAR. The present study cannot estimate whether patients have a benefit of patient-specific TAR implantation.

## References

[1] Glazebrook M Daniels T Younger A , et al. Comparison of health-related quality of life between patients with end-stage ankle and hip arthrosis. J Bone Joint Surg Am. 2008;90:499-505. 18310699 10.2106/JBJS.F.01299

[2] Raikin SM Rasouli MR Espandar R Maltenfort MG. Trends in treatment of advanced ankle arthropathy by total ankle replacement or ankle fusion. Foot Ankle Int. 2014;35:216-224. 24357680 10.1177/1071100713517101

[3] Terrell RD Montgomery SR Pannell WC , et al. Comparison of practice patterns in total ankle replacement and ankle fusion in the United States. Foot Ankle Int. 2013;34:1486-1492. 23774468 10.1177/1071100713494380

[4] LaMothe J Seaworth CM Do HT Kunas GC Ellis SJ. Analysis of total ankle arthroplasty survival in the United States using multiple state databases. Foot Ankle Spec. 2016;9:336-341. 27009079 10.1177/1938640016640891

[5] Kostuj T Preis M Walther M Aghayev E Krummenauer F Roder C . German total ankle replacement register of the German Foot and Ankle Society (D. A. F.)—presentation of design and reliability of the data as well as first results [in German]. Z Orthop Unfall. 2014;152:446-454. 25313699 10.1055/s-0034-1382933

[6] Henricson A Nilsson JA Carlsson A. 10-Year survival of total ankle arthroplasties: a report on 780 cases from the Swedish Ankle Register. Acta Orthop. 2011;82:655-659. 22066551 10.3109/17453674.2011.636678 PMC3247880

[7] Labek G Thaler M Janda W Agreiter M Stockl B. Revision rates after total joint replacement: cumulative results from worldwide joint register datasets. J Bone Joint Surg Br. 2011;93:293-297. 21357948 10.1302/0301-620X.93B3.25467

[8] Labek G Todorov S Iovanescu L Stoica CI Bohler N. Outcome after total ankle arthroplasty-results and findings from worldwide arthroplasty registers. Int Orthop. 2013;37:1677-1682. 23832166 10.1007/s00264-013-1981-7 PMC3764282

[9] Barg A Zwicky L Knupp M Henninger HB Hintermann B. HINTEGRA total ankle replacement: survivorship analysis in 684 patients. J Bone Joint Surg Am. 2013;95:1175-1183. 23824385 10.2106/JBJS.L.01234

[10] Clough T Bodo K Majeed H Davenport J Karski M. Survivorship and long-term outcome of a consecutive series of 200 Scandinavian Total Ankle Replacement (STAR) implants. Bone Joint J. 2019;101-B:47-54. 30601052 10.1302/0301-620X.101B1.BJJ-2018-0801.R1

[11] Saito GH Sanders AE O’Malley MJ Deland JT Ellis SJ Demetracopoulos CA. Accuracy of patient-specific instrumentation in total ankle arthroplasty: a comparative study. Foot Ankle Surg. 2019;25:383-389. 30321969 10.1016/j.fas.2018.02.008

[12] Harston A Lazarides AL Adams SB DeOrio JK Easley ME Nunley JA. Midterm outcomes of a fixed-bearing total ankle arthroplasty with deformity analysis. Foot Ankle Int. 2017;38:1295-1300. 28948831 10.1177/1071100717731853

[13] Gramlich Y Neun O Klug A , et al. Total ankle replacement leads to high revision rates in post-traumatic end-stage arthrosis. Int Orthop. 2018;42:2375-2381. 29560526 10.1007/s00264-018-3885-z

[14] Koo K Liddle AD Pastides PS Rosenfeld PF. The Salto total ankle arthroplasty—clinical and radiological outcomes at five years. Foot Ankle Surg. 2019;25:523-528. 30321952 10.1016/j.fas.2018.04.003

[15] Usuelli FG Maccario C Granata F Indino C Vakhshori V Tan EW. Clinical and radiological outcomes of transfibular total ankle arthroplasty. Foot Ankle Int. 2019;40:24-33. 30203667 10.1177/1071100718798851

[16] Usuelli FG Indino C Maccario C , et al. A modification of the fibular osteotomy for total ankle replacement through the lateral transfibular approach. J Bone Joint Surg Am. 2019;101:2026-2035. 31764365 10.2106/JBJS.19.00307

[17] SooHoo NF Zingmond DS Ko CY. Comparison of reoperation rates following ankle arthrodesis and total ankle arthroplasty. J Bone Joint Surg Am. 2007;89:2143-2149. 17908889 10.2106/JBJS.F.01611

[18] Hauer G Vielgut I Amerstorfer F Maurer-Ertl W Leithner A Sadoghi P. Survival rate of short-stem hip prostheses: a comparative analysis of clinical studies and national arthroplasty registers. J Arthroplasty. 2018;33:1800-1805. 29428465 10.1016/j.arth.2018.01.017

[19] de Steiger RN Liu YL Graves SE. Computer navigation for total knee arthroplasty reduces revision rate for patients less than sixty-five years of age. J Bone Joint Surg Am. 2015;97:635-642. 25878307 10.2106/JBJS.M.01496

[20] Arndt A Westblad P Winson I Hashimoto T Lundberg A. Ankle and subtalar kinematics measured with intracortical pins during the stance phase of walking. Foot Ankle Int. 2004;25:357-364. 15134619 10.1177/107110070402500514

[21] Barnett CH Napier JR. The axis of rotation at the ankle joint in man; its influence upon the form of the talus and the mobility of the fibula. J Anat. 1952;86:1-9. 14907546 PMC1273922

[22] Cho HJ Kwak DS Kim IB. Analysis of movement axes of the ankle and subtalar joints: relationship with the articular surfaces of the talus. Proc Inst Mech Eng H. 2014;228:1053-1058. 25332153 10.1177/0954411914554820

[23] de Asla RJ Wan L Rubash HE Li G. Six DOF in vivo kinematics of the ankle joint complex: application of a combined dual-orthogonal fluoroscopic and magnetic resonance imaging technique. J Orthop Res. 2006;24:1019-1027. 16609963 10.1002/jor.20142

[24] Imai K Tokunaga D Takatori R , et al. In vivo three-dimensional analysis of hindfoot kinematics. Foot Ankle Int. 2009;30:1094-1100. 19912721 10.3113/FAI.2009.1094

[25] Kleipool RP Blankevoort L. The relation between geometry and function of the ankle joint complex: a biomechanical review. Knee Surg Sports Traumatol Arthrosc. 2010;18:618-627. 20300732 10.1007/s00167-010-1088-2

[26] Lapidus PW. Kinesiology and mechanical anatomy of the tarsal joints. Clin Orthop Relat Res. 1963;30:20-36. 4968239

[27] Parr WC Chatterjee HJ Soligo C. Calculating the axes of rotation for the subtalar and talocrural joints using 3D bone reconstructions. J Biomech. 2012;45:1103-1107. 22284429 10.1016/j.jbiomech.2012.01.011

[28] Sancisi N Parenti-Castelli V Corazza F Leardini A. Helical axis calculation based on Burmester theory: experimental comparison with traditional techniques for human tibiotalar joint motion. Med Biol Eng Comput. 2009;47:1207-1217. 19730914 10.1007/s11517-009-0522-4

[29] Seiler H. The upper ankle joint: biomechanics and functional anatomy. Orthopade. 1999;28:460-468. 10.1007/s001320050372 28246996

[30] Sheehan FT. The instantaneous helical axis of the subtalar and talocrural joints: a non-invasive in vivo dynamic study. J Foot Ankle Res. 2010;3:13. 20626876 10.1186/1757-1146-3-13 PMC2912255

[31] Siegler S Toy J Seale D Pedowitz D. The Clinical Biomechanics Award 2013—presented by the International Society of Biomechanics: new observations on the morphology of the talar dome and its relationship to ankle kinematics. Clin Biomech (Bristol, Avon). 2014;29:1-6. 24216466 10.1016/j.clinbiomech.2013.10.009

[32] van den Bogert AJ Smith GD Nigg BM. In vivo determination of the anatomical axes of the ankle joint complex: an optimization approach. J Biomech. 1994;27:1477-1488. 7806555 10.1016/0021-9290(94)90197-x

[33] Barg A Harris MD Henninger HB , et al. Medial distal tibial angle: comparison between weightbearing mortise view and hindfoot alignment view. Foot Ankle Int. 2012;33:655-661. 22995233 10.3113/FAI.2012.0655

[34] Barg A Amendola RL Henninger HB Kapron AL Saltzman CL Anderson AE. Influence of ankle position and radiographic projection angle on measurement of supramalleolar alignment on the anteroposterior and hindfoot alignment views. Foot Ankle Int. 2015;36:1352-1361. 26116431 10.1177/1071100715591091

[35] Choi WJ Kim BS Lee JW. Preoperative planning and surgical technique: how do I balance my ankle? Foot Ankle Int. 2012;33:244-249. 22734289 10.3113/FAI.2012.0244

[36] Berlet GC Penner MJ Lancianese S Stemniski PM Obert RM. Total ankle arthroplasty accuracy and reproducibility using preoperative CT scan-derived, patient-specific guides. Foot Ankle Int. 2014;35:665-676. 24719401 10.1177/1071100714531232

[37] Datir A Xing M Kakarala A Terk MR Labib SA. Radiographic evaluation of INBONE total ankle arthroplasty: a retrospective analysis of 30 cases. Skeletal Radiol. 2013;42:1693-1701. 24026069 10.1007/s00256-013-1718-0

[38] Gotz J Grifka J Springorum HR May S Baier C . Endoprosthetic treatment of the ankle [in German]. Z Rheumatol. 2014;73:788-795. 25315121 10.1007/s00393-014-1404-1

[39] Park JS Mroczek KJ. Total ankle arthroplasty. Bull NYU Hosp Jt Dis. 2011;69:27-35. 21332436

[40] Anderl W Pauzenberger L Kolblinger R , et al. Patient-specific instrumentation improved mechanical alignment, while early clinical outcome was comparable to conventional instrumentation in TKA. Knee Surg Sports Traumatol Arthrosc. 2016;24:102-111. 25326759 10.1007/s00167-014-3345-2

[41] Cavaignac E Pailhe R Laumond G , et al. Evaluation of the accuracy of patient-specific cutting blocks for total knee arthroplasty: a meta-analysis. Int Orthop. 2015;39:1541-1552. 25300397 10.1007/s00264-014-2549-x

[42] Kotela A Kotela I. Patient-specific computed tomography based instrumentation in total knee arthroplasty: a prospective randomized controlled study. Int Orthop. 2014;38:2099-2107. 24968788 10.1007/s00264-014-2399-6

[43] MacDessi SJ Jang B Harris IA Wheatley E Bryant C Chen DB. A comparison of alignment using patient specific guides, computer navigation and conventional instrumentation in total knee arthroplasty. Knee. 2014;21:406-409. 24378337 10.1016/j.knee.2013.11.004

[44] Roh YW Kim TW Lee S Seong SC Lee MC. Is TKA using patient-specific instruments comparable to conventional TKA? A randomized controlled study of one system. Clin Orthop Relat Res. 2013;471:3988-3995. 23907610 10.1007/s11999-013-3206-1 PMC3825894

[45] Tibesku CO Hofer P Portegies W Ruys CJ Fennema P. Benefits of using customized instrumentation in total knee arthroplasty: results from an activity-based costing model. Arch Orthop Trauma Surg. 2013;133:405-411. 23242451 10.1007/s00402-012-1667-4

[46] Hsu AR Davis WH Cohen BE Jones CP Ellington JK Anderson RB. Radiographic outcomes of preoperative CT scan-derived patient-specific total ankle arthroplasty. Foot Ankle Int. 2015;36:1163-1169. 25941196 10.1177/1071100715585561

[47] Daigre J Berlet G Van Dyke B Peterson KS Santrock R. Accuracy and reproducibility using patient-specific instrumentation in total ankle arthroplasty. Foot Ankle Int. 2017;38:412-418. 27920333 10.1177/1071100716682086

[48] Claassen L Luedtke P Yao D , et al. Ankle morphometry based on computerized tomography. Foot Ankle Surg. 2019;25:674-678. 30306892 10.1016/j.fas.2018.08.002

[49] Claassen L Luedtke P Yao D , et al. The geometrical axis of the talocrural joint—suggestions for a new measurement of the talocrural joint axis. Foot Ankle Surg. 2019;25:371-377. 30306891 10.1016/j.fas.2018.02.003

[50] Kellgren JH Lawrence JS. Radiological assessment of osteo-arthrosis. Ann Rheum Dis. 1957;16:494-502. 13498604 10.1136/ard.16.4.494 PMC1006995

[51] Wu G Siegler S Allard P , et al. ISB recommendation on definitions of joint coordinate system of various joints for the reporting of human joint motion—part I: ankle, hip, and spine. International Society of Biomechanics. J Biomech. 2002;35:543-548. 11934426 10.1016/s0021-9290(01)00222-6

[52] Usuelli FG Maccario C Indino C Manzi L Gross CE. Tibial slope in total ankle arthroplasty: anterior or lateral approach. Foot Ankle Surg. 2017;23:84-88. 28578799 10.1016/j.fas.2016.10.001

[53] Usuelli FG D’Ambrosi R Manzi L Maccario C Indino C. Treatment of ankle osteoarthritis with total ankle replacement through a lateral transfibular approach. J Vis Exp. 2018;(131):56396. doi:10.3791/56396. 29443030 PMC5908692

[54] Millington SA Grabner M Wozelka R Anderson DD Hurwitz SR Crandall JR. Quantification of ankle articular cartilage topography and thickness using a high resolution stereophotography system. Osteoarthritis Cartilage. 2007;15:205-211. 16949841 10.1016/j.joca.2006.07.008

[55] Green C Fitzpatrick C FitzPatrick D Stephens M Quinlan W Flavin R. Definition of coordinate system for three-dimensional data analysis in the foot and ankle. Foot Ankle Int. 2011;32:193-199. 21288421 10.3113/FAI.2011.0193

[56] Baxter JR Mani SB Chan JY Vulcano E Ellis SJ. Crossed-screws provide greater tarsometatarsal fusion stability compared to compression plates. Foot Ankle Spec. 2015;8:95-100. 25053793 10.1177/1938640014543358

[57] Tibesku CO Innocenti B Wong P Salehi A Labey L. Can CT-based patient-matched instrumentation achieve consistent rotational alignment in knee arthroplasty? Arch Orthop Trauma Surg. 2012;132:171-177. 22006572 10.1007/s00402-011-1406-2

[58] Nogler M Hozack W Collopy D Mayr E Deirmengian G Sekyra K. Alignment for total knee replacement: a comparison of kinematic axis versus mechanical axis techniques: a cadaver study. Int Orthop. 2012;36:2249-2253. 22890847 10.1007/s00264-012-1642-2 PMC3479289

[59] Siegler S Chen J Schneck CD. The three-dimensional kinematics and flexibility characteristics of the human ankle and subtalar joints—part I: kinematics. J Biomech Eng. 1988;110:364-373. 3205022 10.1115/1.3108455

[60] Siegler S Udupa JK Ringleb SI , et al. Mechanics of the ankle and subtalar joints revealed through a 3D quasi-static stress MRI technique. J Biomech. 2005;38:567-578. 15652556 10.1016/j.jbiomech.2004.03.036

[61] Lundberg A Svensson OK Nemeth G Selvik G. The axis of rotation of the ankle joint. J Bone Joint Surg Br. 1989;71:94-99. 2915016 10.1302/0301-620X.71B1.2915016

[62] Richter M Zech S Westphal R Klimesch Y Gosling T. Robotic cadaver testing of a new total ankle prosthesis model (German Ankle System). Foot Ankle Int. 2007;28:1276-1286. 18173992 10.3113/FAI.2007.1276

